# Recent and Advanced DNA-Based Technologies for the Authentication of Probiotic, Protected Designation of Origin (PDO) and Protected Geographical Indication (PGI) Fermented Foods and Beverages

**DOI:** 10.3390/foods12203782

**Published:** 2023-10-14

**Authors:** Vincenzina Fusco, Francesca Fanelli, Daniele Chieffi

**Affiliations:** Institute of Sciences of Food Production, National Research Council of Italy (CNR-ISPA), 70126 Bari, Italy; francesca.fanelli@ispa.cnr.it (F.F.); daniele.chieffi@ispa.cnr.it (D.C.)

**Keywords:** food authenticity, probiotic food and beverages, fermented food, metagenomics, propidium monoazide (PMA) real-time PCR

## Abstract

The authenticity of probiotic products and fermented foods and beverages that have the status of protected designation of origin (PDO) or geographical indication (PGI) can be assessed via numerous methods. DNA-based technologies have emerged in recent decades as valuable tools to achieve food authentication, and advanced DNA-based methods and platforms are being developed. The present review focuses on the recent and advanced DNA-based techniques for the authentication of probiotic, PDO and PGI fermented foods and beverages. Moreover, the most promising DNA-based detection tools are presented. Strain- and species-specific DNA-based markers of microorganisms used as starter cultures or (probiotic) adjuncts for the production of probiotic and fermented food and beverages have been exploited for valuable authentication in several detection methods. Among the available technologies, propidium monoazide (PMA) real-time polymerase chain reaction (PCR)-based technologies allow for the on-time quantitative detection of viable microbes. DNA-based lab-on-a-chips are promising devices that can be used for the on-site and on-time quantitative detection of microorganisms. PCR-DGGE and metagenomics, even combined with the use of PMA, are valuable tools allowing for the fingerprinting of the microbial communities, which characterize PDO and PGI fermented foods and beverages, and they are necessary for authentication besides permitting the detection of extra or mislabeled species in probiotic products. These methods, in relation to the authentication of probiotic foods and beverages, need to be used in combination with PMA, culturomics or flow cytometry to allow for the enumeration of viable microorganisms.

## 1. Introduction

Aimed at achieving an economic gain, food labeling or food products may be tampered with, substituted or misrepresented, thus committing food fraud. To overcome this problem, which causes an estimated burden of USD 10–15 billion per year on consumers and industry [1], food authentication, which is an analytical process that ascertains label information about the content, the origin and the production process of food, is crucial. 

As stated in the consensus statement on fermented foods by the expert panel of the International Scientific Association for Probiotics and Prebiotics (ISAPP), “fermented foods are foods made through desired microbial growth and enzymatic conversions of food components” [2]. The European Union schemes of geographical indications, known as protected designation of origin (PDO) and protected geographical indication (PGI), promote and protect names of agricultural products and foodstuffs [3]. Products that have PDO or PGI status may be marked with the relevant logo to help identify those products [3]. The schemes are based on the legal framework provided by the EU Regulation No. 1151/2012 of the European Parliament and of the Council of 21 November 2012 on quality schemes for agricultural products and foodstuffs [4]. These schemes protect the name of a product that comes from a specific region and follows a particular traditional production process. In the case of PDOs, the raw ingredients must come from the region of origin where the production process need to take place, whereas for PGIs, at least one of the stages of production, processing or preparation takes place in a specific geographical area [5]. Many fermented cheeses such as the Italian Grana Padano and Parmigiano Reggiano are PDOs [6], while other fermented sausages such as salame felino and salame Cremona are PGIs [7], and numerous fermented beverages such as various Italian wines have PDO or PGI status [8]. 

Each fermented food and beverage is characterized by a peculiar microbiota originating from the raw materials, equipment and processing environment, whose composition and evolution is affected by biotic and abiotic factors that intervene during each specific process of production. Such a microbial consortium ensures both the primary fermentation activities, which guarantee the technological results of the transformation, and the accessory ones, which contribute notably to the definition of the characteristics of typicality and quality, bases of their notoriety. Different microbial fingerprints have been found to be specific to the geographic area of origin [9]. For this reason, microbial fingerprints are used as authenticity markers for the authentication of PDO and PGI fermented foods and beverages [10], whereas for starter cultures or adjuncts, specific amplicons might be used as biomarkers for the authentication of probiotic and fermented foods and beverages [11,12,13,14]. 

But while in the case of fermented foods and beverages, only the detection of either microorganisms used as starter cultures/adjuncts or the whole microbiota is satisfactory, in the specific case of probiotic foods and drinks, the quantification of each probiotic mentioned in the label is also mandatory [12,13,14]. Indeed, according to the latest universally accepted definition by the FAO/WHO (Food and Agriculture Organization of the United Nations/World Health Organization), probiotics are “live microorganisms that, when administered in adequate amounts, confer a health benefit on the host” [15]. Thus, to authenticate a probiotic product, the number of viable cells of each probiotic microorganism claimed in the label, whose identity should be confirmed at both species and strain level, has to be enumerated [12,13,14].

To date, several methods are available to qualitatively and quantitatively detect microbial markers of authenticity in fermented and probiotic foods and beverages. Among these, DNA-based methods, mainly due to their speed, robustness, sensitivity and specificity, are gaining ground in recent decades.

Herein, we provide a review of the advanced DNA-based techniques currently in use or that can be used in the near future to evaluate the authenticity of fermented and probiotic foods and beverages.

## 2. Methods

### 2.1. Polymerase Chain Reaction (PCR)-Based Technologies

Since 1983, when the scientists of the Cetus Corporation of Emerville (CA, USA) [16] launched the PCR, for which Kary Mullis [17] obtained the Nobel Prize, foodborne microorganism diagnostics was revolutionized. This revolutionary DNA-based method requires the use of a thermostable DNA polymerase, which, in presence of two primers, i.e., DNA fragments with a sequence complementary to the extremities of the DNA fragment to be amplified, synthetizes new strands of DNA complimentary to the target sequence [18]. Partial or fully PCR-based amplification and sequence analysis of the 16S rRNA gene has been widely used to identify technological and probiotic bacteria. The authenticity of probiotic supplements and beverages was, for example, assessed by Ansari et al. [19] using 16S rRNA gene sequencing combined with matrix-assisted laser desorption ionization coupled to time-of-flight mass spectrometry (MALDI TOF MS) and phenotypical typing. However, besides being labor- and time-consuming, this method may not allow for the reliable identification of phylogenetically closely related species [14]. PCR assays targeting species-specific regions of genes (Table 1) other than 16S rRNA genes have sped up the identification process and made it more reliable. Moreover, while 16S gene sequencing analysis requires a first step of isolating the target bacterium prior to DNA extraction, the species-specific PCR can be culture-independent, i.e., performed on the DNA directly extracted from the food. In Table 1 it is listed an excerpt of simplex and multiplex PCR assays to detect the main technological and probiotic microorganisms used as starter cultures or adjuncts for the production of probiotic [12,13,14] and PDO and PGI fermented foods and beverages [11,20].

### 2.2. PCR-Based Typing Methods and Whole Genome Sequencing

The authenticity of a technological or probiotic microorganism should be determined not only at the species level but also at the strain level, as stated in the guidelines proposed by the Council for Responsible Nutrition and International Probiotics Association [32], as well as by the Food and Agriculture Organization of the United Nations and the World Health Organization [33].

Random amplified polymorphic DNA (RAPD)-PCR, which employs an oligonucleotide primer to amplify anonymous DNA providing complex strain-specific banding patterns, and amplified fragment length polymorphism (AFLP)-PCR, which combines the restriction endonuclease of the genomic DNA with a PCR involving adaptor- and restriction site-specific primers, are genotyping techniques providing polymorphic banding patterns that allow for strain discrimination [34]. However, restriction endonuclease analysis using pulsed-field gel electrophoresis (REA-PFGE), through which the fragments obtained by the restriction endonuclease of intact genomic DNA extracted and purified in situ are resolved via PFGE, avoids the intrinsic bottleneck of PCR through providing polymorphic banding patterns, referred to as pulsotypes, and is considered the “gold standard” for genotyping [34].

RAPD-PCR, AFLP-PCR and REA-PFGE have been applied to ascertain the authenticity of probiotic foods [35,36,37], the presence of the starter culture in the intermediate and final fermented products [23,38,39] or the correctness of the fermentation process in the production of fermented foods [23,36,40]. However, these methods are time- and labor-consuming. 

Whole genome sequencing (WGS) allows the identification of a target microorganism at both species and strain level. Furthermore, WGS data analysis provides a preliminary in silico assessment of microbial safety and probiotic potential [41,42], evaluating the presence of antimicrobial and biocide resistance genes, as well as genes related to probiotic activities [10,43,44,45,46,47,48,49,50,51,52,53,54,55,56,57,58,59,60,61,62,63,64,65,66,67,68,69]. Such a genome-based approach, referred to as “probiogenomics”, allows us to identify and predict the health-promoting and safety activities of promising probiotic strains [70]. Moreover, through analyzing whole genomes, it is possible to detect strain-specific fragments that can be used to design primers and probes for real-time PCR assays, allowing for the on-time identification and quantification of target microbial strains in fermented and probiotic foods.

### 2.3. Real-Time PCR-Based Methods

Authentic is any probiotic food or supplement whose content is consistent with that claimed on its label [12,13,14].

Assessing the authenticity of a probiotic food is crucial and requires verifying not only the presence and viability of the labeled probiotic but also the labeled amount of viable cells of the probiotic. The gold-standard method to quantify microorganisms is plating. However, plating is time- and labor-consuming and requires more days to achieve results. Moreover, the selective media used to enumerate microorganisms do not allow the enumeration of microbes in the viable but non-culturable (VBNC) state. To overcome this drawback, methods that combine the use of selective and non-selective media in one single system, such as the Lutri plate, the Overlay (OV), the membrane-transferring surface-plating and the thin agar layer (TAL) methods, firstly proposed to detect VBNC pathogenic bacteria, can be used [34,71]. Using these methods, the resuscitation of non-culturable cells is allowed prior to the diffusion of the selective compounds or dyes, which is controlled over time [71]. But even these methods are laborious and require days to produce results [34,71]. Using flow cytometry (FCM) with appropriate fluorescent probes may allow for the quantitative detection of VBNC microorganisms based on the direct examination of specific cell functions [72].

Real-time PCR, which is rapid, robust, sensitive and user-friendly, besides being high-throughput and multiplexing, is being widely applied to investigate the authenticity of probiotic and fermented foods. Using this technique, it is possible to monitor the emission of fluorescence by DNA-intercalating agents or labeled probes at each amplification cycle, which, being proportional to the DNA present in the reaction mixture, allows the real-time quantification of the target DNA and, thus, of the amount of the target microorganism. However, although more sensitive, intercalating dyes are not as specific as labeled probes. This drawback may be overcome by using the melting or the high-resolution melt (known as either hi-res melting or HRM) curve analysis of the obtained amplicons [73]. To make the melting curve analysis, at the end of the real-time PCR run, the thermal cycler starts at a preset temperature (usually above the primer Tm, e.g., 65 °C) and measures the amount of fluorescence. The temperature of the sample is then increased incrementally as the instrument continues to measure fluorescence. As the temperature increases, double-stranded DNA denatures, becoming single-stranded, and the dye dissociates, resulting in decreasing fluorescence. The change in fluorescence is plotted as a function of temperature to obtain the melt curve. The melting temperature (Tm) of an amplicon is defined as “the temperature at which the steepest decrease of signal occurs and correspond to the peak value(s) in the negative derivative of the melting curve” [74]. Each fragment of double-stranded DNA has a melting point (Tm) at which temperature 50% of the DNA is single-stranded. The melting temperature of the amplicon is a function of the length of DNA, guanine–cytosine content, sequence order and Watson–Crick pairing [74]. As soon as the double-stranded DNA containing the binding dye is heated, a decrease in fluorescence is detected when Tm is reached due to the release of dye from the dissociated DNA strands. This point is determined from the inflection point of the melting curve or the melting peak of the derivative plot, i.e., the negative first derivative of the melting curve. Technological advancements enhanced the potential of this technology. In particular, the so-called saturating DNA dyes have significantly increased the specificity and sensitivity of this technology, whereas advanced instruments for measuring melting behavior allowed users to improve the temperature precision combined with increased measurement per time unit and drop in temperature. The resulting so-called high-resolution melting curve analysis (HRM or HRMA) has begun to offer higher sensitivity for single-nucleotide polymorphism (SNP) detection within an entire dye-stained amplicon [75]. Also, droplet digital PCR, which is based on the fractionation of the sample into 20,000 droplets so that PCR amplification of the template molecules occurs in each individual droplet, allows for the absolute quantification of targets with a high sensitivity, and without the use of the standard curve [76]. Species- and even strain-specific primers and probes have been designed to allow the real-time PCR HRM- and ddPCR-based identification of technological and probiotic microorganisms and the authentication of probiotic and fermented foods [77,78,79,80,81,82,83,84,85,86,87,88,89,90,91,92]. 

However, as stated above, DNA-based methods do not discriminate among viable or dead microorganisms, discrimination that can be achieved using culture-based approaches or flow cytometry methodologies [36,72,93]. Propidium monoazide (PMA), with its ability to penetrate compromised cell walls and membranes, once photoactivated, covalently binds the DNA of dead and damaged cells; the resulting monoadduct is not amplifiable. PMA–real-time PCR allows for the on-time quantitative detection of only viable microorganisms and is a diagnostic tool widely applied for the authentication of probiotic and fermented foods [94,95,96,97,98].

### 2.4. DNA-Based Biosensors and Lab-on-a-Chip Devices

Among the numerous diagnostic methods currently available to detect technological and probiotic microorganisms in food, biosensors are promising tools capable of providing high levels of faster and more automated surveillance. These analytical devices combine a sensitive biological element (the receptor) with a chemical or physical transducer to selectively and quantitatively detect the presence of a specific compound in a given environment [99]. The selectivity of the biosensor is determined by the integrated biological component: even in complex matrices (food, tissues, etc.), only certain substances that are capable of interacting with the biological part will be able to generate the electrical, chemical, optical or mechanical signal of the transducer, modulating the selectivity of the biosensor [99]. The intrinsic properties of DNA, such as its high specificity and the possibility of optical, electrical or mechanical detection, make it an excellent candidate for use in this type of application [100]. Given the inherent robustness of PCR and the high sensitivity that can be achieved through the amplification process, PCR-based biosensing is highly used. For PCR-based biosensors, the biological amplification of DNA is exploited, which can be translated into a measurable signal using physical or chemical transducers [101]. In the case of biosensors based on real-time PCR, the fluorescence emission constitutes the measurable signal that allows the transduction of the biological amplification process [34]. In fact, real-time PCR fluorophores are used to directly monitor the amplification of the target DNA. Furthermore, since fluorescence increases proportionally to the logarithm of the amount of amplicon obtained, real-time PCR can be used for the quantitative detection of microorganisms.

The rapid evolution of micro- and nano-technologies has opened new horizons towards the integration and miniaturization of conventional sensing platforms, resulting in the so-called lab-on-a-chip devices that incorporate various laboratory processes in a miniaturized and semi-automated system. However, PCR requires a thermal cycler instrument, which significantly limits the potential miniaturization of the system [101]. To overcome this limit, isothermal amplification methods are being developed [102] and integrated into microfluidic devices [103]. 

The development and use of these integrated platforms for the detection and quantification of microorganisms are still limited to the detection of pathogenic microorganisms [104].

The obvious advantages of these integrated and miniaturized technologies for the detection of technological and probiotic microorganisms lie in the reduction in the volume of reagents used and, therefore, the associated costs, as well as the reduction in time to results. Furthermore, the possibility of making the entire detection system portable and automated facilitates its use not only in the agri-food sector but also in other strategic sectors such as anti-fraud services, port and border control authorities, certification agencies/bodies of control of consortia for the protection of typical products, and public and private control laboratories.

### 2.5. PCR-DGGE 

PCR amplification of variable regions of the 16S rRNA gene (for bacteria) or 18S or 26S rRNA gene (for yeasts and fungi) followed by denaturing gradient gel electrophoresis (PCR-DGGE) is another technique frequently used in recent decades to investigate the authenticity of probiotic and fermented foods and beverages. This PCR-based method is based on the separation, in a denaturing gradient gel, of amplicons of the same size but with different sequences, based on the differential denaturation (melting) profile [105]. PCR-DGGE results in a pattern of amplicons that can allow for the identification of the species either (i) through comparing the position of a band to a reference, (ii) through the sequence analysis of the band eluted from the gel or (iii) through band hybridization. This technique has allowed the authentication of probiotic [37,106,107,108,109,110] and fermented [39,111,112] foods. Moreover, the pattern of amplicons obtained via PCR-DGGE, which theoretically reflect the microbial community of the target food, can be specific to the geographic area of origin, thus allowing to ascertain the authenticity of PDO and PGI foods and beverages and the traceability of these products [113,114,115,116,117,118,119,120,121,122,123]. However, PCR-DGGE has drawbacks such as, for example, preferential PCR, the formation of chimeric or heteroduplex molecules and the co-migration of different amplicons [39,105,111,124].

### 2.6. Metagenetics and Metagenomics

Based on the same principle as PCR-DGGE, 16S metagenetic analysis and metagenomics may allow the characterization of fermented foods and the authentication of PDO and PGI products as well as that of probiotic foods. Metagenetics, also called amplicon sequencing, metabarcoding, metataxonomics, 16S metagenomics (targeting bacteria), 18S or 23S or ITS (internal transcribed spacer) metagenomics (targeting fungi), consists of the PCR amplification of target genes from metagenomic DNA combined with sequencing and alignment against a reference database to detect the microbial composition of a microbial community [125].

As an example of using this technology to describe the microbial community of foods, Celano et al. [126], via 16S metagenetic analysis, showed that *Lactobacillus helveticus* dominated both natural whey starter cultures and the corresponding Caprino and Vaccino cheeses, two traditional cheeses produced in the same dairy farm, whereas *Staphylococcus equorum* and *Streptococcus thermophilus* dominated Cacioricotta and Pecorino cheeses, respectively, also produced in the same dairy plant. The parallel assessment of the enzymatic activities, degree of proteolysis, and concentrations of the main compounds involved in the sensory traits of these 4 traditional cheeses produced in the same dairy plant allowed the authors to highlight the distinctive features of these cheeses and to find the relationships between their microbiological and biochemical characteristics [126]. Also, 16S metagenetics has been used to assess the authenticity of probiotic foods [14]. For example, Ullah et al. [127] combined culture-independent 16S metagenetics with culture-dependent 16S metagenetics (applied to DNA extracted from colonies plated on selective agar) to assess the authenticity of 19 probiotic products, whereas Shehata and Newmaster [87] used a polyphasic approach combining cultivation, species- and/or strain-specific PCRs with culture-independent 16S metabarcoding for the authentication of 182 probiotic products.

However, metagenetics has drawbacks linked to the low discriminatory power of ribosomal RNA to identify phylogenetically closely related bacteria and fungi, apart from the biases of PCR amplification [36]. These problems can be overcome via shotgun metagenomics, commonly known as metagenomics, which is an untargeted method that sequences the entire DNA sample extracted from a microbial population [125]. Using this method, the sample DNA is fragmented, and a library is prepared and sequenced so that the resulting data allow one to obtain information on the taxonomic composition of the microbial population [125]. If the method of extraction of the genomic DNA from a microbial community is efficient, metagenomics provides information not only about bacteria but also about viruses, archaea and single-celled eukaryotes like fungi [125]. Moreover, metagenomics may provide metagenome-assembled genomes that can also furnish more genomic information at strain level [125]. Shotgun metagenomics is emerging as a reliable tool to assess the authenticity of probiotic and fermented foods [128,129,130]. However, this untargeted technique is more complex and expensive, necessitate cumbersome and complex equipment as well as advanced bioinformatic skills, and requires more time to get results. Nevertheless, this technique has the advantage of providing information on the microbiome and the single genomes present in a food or beverage matrix. If combined with culturomics, flow cytometry or the use of PMA, it may result in the characterization of the viable microbial community present in the product [131].

## 3. Concluding Remarks

**Figure 1:**
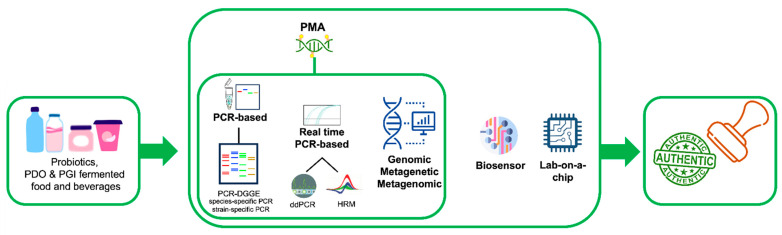
DNA-based technologies for the authentication of probiotic products, PDO and PGI fermented foods and beverages.

DNA-based technologies are valuable tools to assess the authenticity of probiotic, PDO and PGI (fermented) foods and beverages. Several DNA-based methods are presently available (Figure 1), but, depending on the specific goal, the available equipment and the budget, the most adequate should be chosen each time. If the objective is to assess the authenticity of probiotic and fermented foods and beverages, PMA isothermal amplification-based lab-on-a-chip devices may represent valuable and appropriate tools that may allow the on-site and on-time qualitative–quantitative detection of viable foodborne and probiotic microorganisms. However, although such devices are very promising, they are currently used only for pathogenic microorganism detection. Thus, the assessment of the efficacy of these devices in the quali/quantitative detection of probiotic and starter cultures represents a fruitful avenue for future work.

PMA real-time PCR-based assays, being a multiplexed and high-throughput method, allow for the simultaneous qualitative–quantitative detection of numerous probiotics or starter cultures directly from different food and beverage matrices. However, the primers and probes should be strain-specific to allow for the qualitative–quantitative detection of the labeled probiotic strain or starter culture. In this context, the pangenome-based design of primers and probes specific to each target strain is worthwhile.

If the necessity is to investigate the presence of extra species or of mislabeled species in probiotic food and beverages or to assess the authenticity of PDO and PGI fermented foods and beverages, it is necessary to use the whole microbiota as an authenticity marker. In this case, depending on the budget and equipment available, metagenomics or PCR-DGGE can be used. But while the sole microbial pattern of PDO and PGI fermented foods and beverages allows for their authentication, in the case of probiotic food and beverages, a quantitative detection method for viable microbes such as the use of PMA in combination with the above-mentioned methods or the employment of culturomics or flow cytometry in combination with these community fingerprinting techniques is mandatory.

## Figures and Tables

**Table 1:** Species-specific PCR for the main technological and/or probiotic microorganisms.

Species	Target Gene	Product Encoded by the Gene	References
*Streptococcus thermophylus*	*lac*Z	Β-galactosidase enzyme	[21]
*Lactobacillus delbrueckii* subsp. *bulgaricus*	*tuf* 16S rRNA	elongation factor Tu 16S rRNA	[22]
*Levilactobacillus brevis*	Gene encoding the aldo/keto reductase of the diketogulonate-reductase family of *L. brevis*	aldo/keto reductase of the diketogulonate-reductase family of *L. brevis*	[23]
*Weissella confusa*	*lep*A gene	GTPbinding protein LepA (Elongation Factor 4)	[24]
*Lacticaseibacillus casei*	16S rRNA	Ribosomal RNA	[25]
*Lacticaseibacillus paracasei*			
*Lacticaseibacillus rhamnosus*			
*Lactobacillus helveticus*	*pep*C *pep*N *htr*A	aminopeptidases C aminopeptidase N, trypsin-like serine protease	[26]
*Limosilactobacillus fermentum*	16S rRNA	Ribosomal RNA	[27]
*Lactiplantibacillus plantarum* *Lactiplantibacillus pentosus* *Lactiplantibacillus paraplantarum*	*rec*A	RecA	[28]
37 *Lactobacillus* species		16S-23S rRNA gene	[29]
*Leuconostoc (Ln*.) *mesenteroides*, *Ln. pseudomesenteroides*, *Ln. lactis* and *Ln. citreum*		*Hsp*60	[30]
*Enterococcus (E.) faecalis*, *E. faecium*, *E. hirae*, and *E. casseliflavus*		58 specific molecular targets obtained by pan-genome analysis	[31]

## Data Availability

The data used to support the findings of this study can be made available by the corresponding author upon request.
